# Modality redundancy for MRI-based glioblastoma segmentation

**DOI:** 10.1007/s11548-024-03238-4

**Published:** 2024-08-02

**Authors:** Selene De Sutter, Joris Wuts, Wietse Geens, Anne-Marie Vanbinst, Johnny Duerinck, Jef Vandemeulebroucke

**Affiliations:** 1 Department of Electronics and Informatics (ETRO), Vrije Universiteit Brussel (VUB), Brussels, Belgium; 2 Department of Neurosurgery, Universitair Ziekenhuis Brussel (UZ Brussel), Vrije Universiteit Brussel (VUB), Brussels, Belgium; 3 Department of Radiology, Universitair Ziekenhuis Brussel (UZ Brussel), Vrije Universiteit Brussel (VUB), Brussels, Belgium; 4 imec, Leuven, Belgium; 5 Department of Radiology and Medical Imaging, Cliniques Universitaires Saint Luc, Université Catholique de Louvain (UCLouvain), Brussels, Belgium

**Keywords:** Glioblastoma, Segmentation, Uncertainty, Magnetic resonance imaging (MRI), Deep learning, BraTS

## Abstract

**Purpose:**

Automated glioblastoma segmentation from magnetic resonance imaging is generally performed on a four-modality input, including T1, contrast T1, T2 and FLAIR. We hypothesize that information redundancy is present within these image combinations, which can possibly reduce a model’s performance. Moreover, for clinical applications, the risk of encountering missing data rises as the number of required input modalities increases. Therefore, this study aimed to explore the relevance and influence of the different modalities used for MRI-based glioblastoma segmentation.

**Methods:**

After the training of multiple segmentation models based on nnU-Net and SwinUNETR architectures, differing only in their amount and combinations of input modalities, each model was evaluated with regard to segmentation accuracy and epistemic uncertainty.

**Results:**

Results show that T1CE-based segmentation (for enhanced tumor and tumor core) and T1CE-FLAIR-based segmentation (for whole tumor and overall segmentation) can reach segmentation accuracies comparable to the full-input version. Notably, the highest segmentation accuracy for nnU-Net was found for a three-input configuration of T1CE-FLAIR-T1, suggesting the confounding effect of redundant input modalities. The SwinUNETR architecture appears to suffer less from this, where said three-input and the full-input model yielded statistically equal results.

**Conclusion:**

The T1CE-FLAIR-based model can therefore be considered as a minimal-input alternative to the full-input configuration. Addition of modalities beyond this does not statistically improve and can even deteriorate accuracy, but does lower the segmentation uncertainty.

## Introduction

Glioblastoma (GBM) is a malignant type of brain tumor that originates in the glial cells of the brain [1]. It is typically diagnosed by magnetic resonance imaging (MRI), on which it can be considered to consist of three components: the contrast-enhanced (CE) part, central necrosis (NEC), and the adjacent non-contrast-enhancing component, including both infiltrating tumor and vasogenic edema (ED) [2]. Accurate segmentation of these structures is valuable for therapy planning, including surgery and radiotherapy [3]. Manual delineation, however, is time-consuming and prone to inter-reader variability.

Consequently, an extensive amount of studies have been dedicated to the automated segmentation of GBM from medical imaging data [4–6]. Since 2012, the yearly Brain Tumor Segmentation (BraTS) challenge [2, 7, 8] has been providing an increasingly large data set with three-dimensional expert-annotated MRI of GBM with the purpose of developing such methods. The data set includes pre- and post-contrast T1-weighted (T1 and T1CE), T2-weighted (T2) and fluid attenuated inversion recovery (FLAIR) sequences for all subjects, as these encompass the standard GBM imaging protocol [9, 10].

The described MRI modalities can be obtained through changes in the transverse and longitudinal relaxation times [5, 11] and capture complementing information [6]. While T1 imaging reveals tissue anatomy and structure [6], T1CE arises from a similar acquisition with the addition of a contrast-agent, typically gadolinium [5]. The contrast highlights areas where the blood–brain barrier is disrupted, indicating the dense presence of tumor cells [12]. T2 images are sensitive to variations in water content, making them suited for the visualization of edema and fluid-filled structures. The acquisition of FLAIR is similar to T2, but is followed by an inversion recovery pulse sequence, which is designed to suppress the signal from cerebrospinal fluid (CSF), facilitating its differentiation from hyperintense abnormalities [5, 6, 10, 12]. While FLAIR and T2 hyper-intensities can reveal the edema region, T1 and T1CE typically visualize the tumor core [2, 6, 13].

The nature of these acquisitions lead us to hypothesize that redundancy might be present in these images. There might be considerable overlap in the information, referred to as *duplicative* redundancy, and not all information might be essential for tumor segmentation, which we describe as *irrelevant* redundancy. Indeed, saliency maps, which highlight image regions that are important for a model’s prediction, generated from a glioma segmentation network in [14] reveal discrepancies in the importance of the different modalities, suggesting the presence of redundancy for this distinct task.

While one could assume redundancy could enhance result robustness, a general rule in machine learning states that larger numbers of features and a correspondingly higher dimensionality can increase chances of overfitting, raising the prediction error when this is not countered with more training examples [15–17]. Even more, a study by D’Amario et al. [18] investigating the influence of input dimensions on the performance of deep learning networks concluded that input redundancy can effectively decrease the performance of the model. However, the effect appears to be dependent on whether the redundancy is duplicative, irrelevant or both, raising the question on how this translates to possible redundancy in MR modalities for glioma segmentation.

Furthermore, in clinical practice, some of these modalities are regularly missing due to reasons such as scanning time and costs, scanner availability and patient comfort, as well as noise and artifacts that have corrupted the images [9, 19–21]. The development of automatic GBM segmentation models relies predominantly on 3D imaging. It has been demonstrated to exhibit advantages compared to 2D imaging, including improvements in appreciation of anatomical location, longitudinal assessment, measurement of tumor volume and detection of small lesions [9]. However, a survey in 2018 [9] covering 220 institutions in 31 European countries showed that although 2D acquisitions of all four modalities were included in routine imaging for 95.5% of the cases, this is not true for 3D acquisitions due to reasons such as time pressure, scanner limitations, financial considerations, quality concerns or a lack of technical support. The study reports the routine acquisition of 3D T1CE in 98.3% of the cases, yet only 31.1% for FLAIR, 9.4% for T1 and 3.9% for T2, indicating the ubiquity of missing 3D modalities in clinical practice.

A large number of studies strove to solve the missing modality issue for GBM segmentation on MRI [21, 22]. Unfortunately, these solutions often provide complex networks associated with elaborate training methods [22], therefore requiring more data samples and increasing their risk of overfitting [23].

While the conducted studies mostly focus on missing data during testing and assume the presence of the whole MR-spectrum during training [21], exploiting only the essential modalities for both stages is accompanied by a range of advantages. In addition to reducing the necessary acquisition time and related costs by directly facilitating inference using fewer modalities, it also would allow the retrospective inclusion of subjects which have missing modalities and would otherwise have to be excluded. This enables an expansion of study data, where the amount of samples is of vital importance, especially in a data-scarce medical context.

This reasoning encourages us to rephrase the question from “How can we complement missing modalities?”, to “Which modalities are essential for accurate automated GBM segmentation?”. In this study, we therefore aim to explore the importance and influence of the MR modalities for the automatic segmentation of GBM. To the best of our knowledge, this research explores a novel perspective with no prior precedent.

**Fig. 1:**
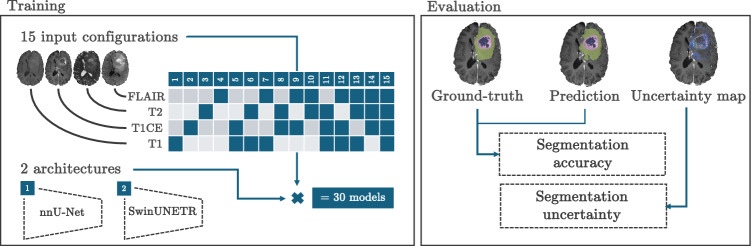
Summary of the workflow. On the left, an overview of the different model configurations is shown, including 15 input configurations and two architectures, resulting in a total of 30 models. Modalities incorporated into a model are highlighted in dark in the table. After training, each of the models is evaluated in terms of segmentation accuracy and uncertainty. Colors of segmentation refer to ED (green), CE (pink) and NEC (blue)

**Fig. 2:**
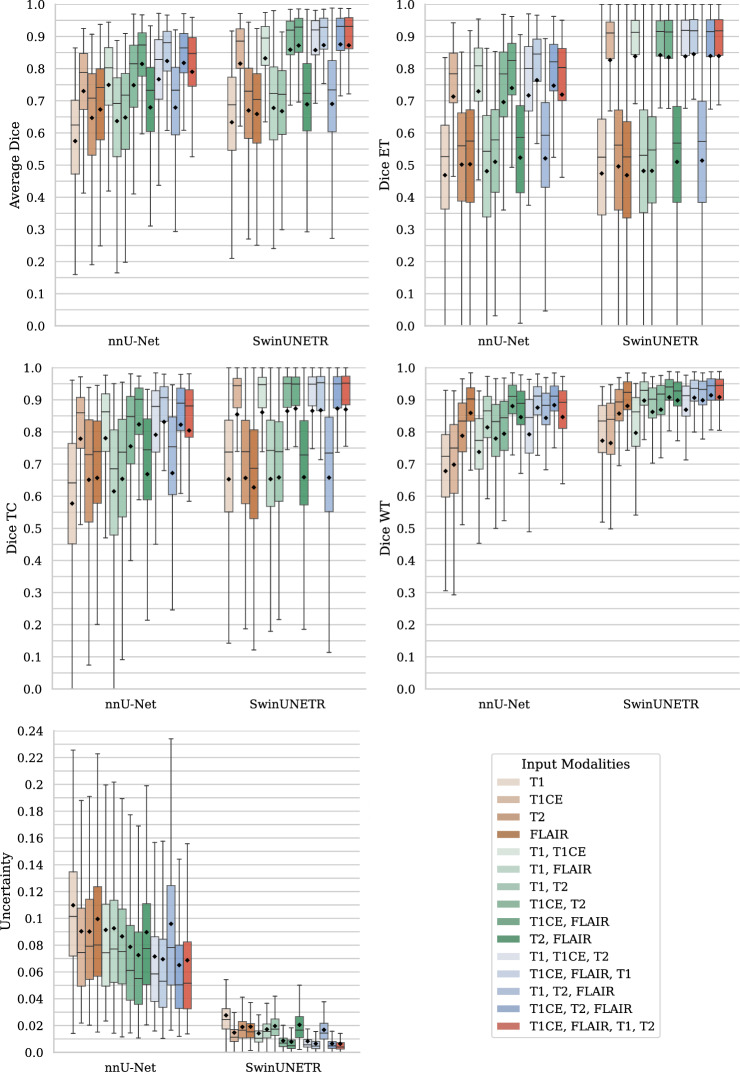
Boxplots of Dice scores overall (upper left), for enhanced tumor (upper right), for tumor core (middle left) and for whole tumor (middle right) and uncertainty scores (lower left) for different input modality combinations. Color hues correspond to single-input (orange), two-input (green), three-input (blue) and full-input (red) configurations. Mean values are denoted by the diamond marker

## Methods

### Data and preprocessing

Data from the BraTS21 challenge’s  [2, 7, 8] original training set was used, including 1251 GBM subjects, each containing four MRI scans (T1, T1CE, T2 and FLAIR) and expert-annotated ground-truth segmentations for the CE, NEC and ED region. The data set was randomly split further into a training (70%) and test (30%) set. Images in the BraTS data set are co-registered, resampled to an isotropic voxel spacing of 1 mm, and skull-stripped [24–26]. Additionally, image intensities were normalized using Z-scoring.

### Model training

Two 3D model architectures were included for comparison. The first model consists of the full-resolution model as defined in the nnU-Net framework [27], considered the state of the art among CNNs for segmentation. Recently, transformers are gaining popularity for segmentation tasks. Therefore, a SwinUNETR [28] model was included as well. Both architectures were trained in a similar fashion on the training set for 1000 epochs. The patch and batch size were (128, 128, 128) and 4, respectively. Cross-entropy Dice loss was used as loss function. An initial learning rate of 1e$-4$ with a cosine annealing schedule was combined with an Adam optimizer and a 1e-5 weight decay. A dropout rate of 0.2 was used. Data augmentation included random flipping along all three axis, as well as random intensity scaling and shifting.

Generally, GBM segmentation is performed using a four-channel input (T1, T1CE, T2 and FLAIR). In this study, different models were trained with varying input channels, differing by their amount and combinations of input modalities. Along with the two model architectures, this gave rise to a total of 30 segmentation models (Fig. 1).

The resources and services used in this work were provided by the VSC (Flemish Supercomputer Center), funded by the Research Foundation—Flanders (FWO) and the Flemish Government. All models were trained on an Nvidia A100 Ampere GPU with 40 GB RAM together with a 16-core AMD EPYC 7282 CPU. MONAI version 1.2.0 [29] was used for model implementations. The detailed implementation for training and all trained models developed in this work are made publicly available.^1^

### Model evaluation

#### Segmentation accuracy

Each model was evaluated in terms of segmentation performance on the test set through calculation of the Dice similarity coefficient between the predicted and ground-truth segmentation, according to formula 1.

1 $$\begin{aligned} \textrm{Dice} = \frac{2*\textrm{TP}}{2* \textrm{TP} + \mathrm FP + \mathrm FN} \,, \end{aligned}$$

where TP is the amount of true positive voxels, FP is the amount of false positive voxels and FN is the amount of false negative voxels.

The prediction consists of three labels: necrosis (NEC), contrast-enhancing tumor (CE) and edema (ED). Consequently, Dice scores were calculated over three regions: enhanced tumor (ET = CE), tumor core (TC = CE + NEC) and whole tumor (WT = CE + NEC + ED). An overall Dice score was obtained by averaging the Dice over these three regions.

Subsequently, segmentation accuracies of all configurations are compared to the full-input configuration. From this, we define the minimal-input configuration as the configuration with the fewest amount of modalities that yields similar performance as the full-input configuration. Analogously, we define the optimal performance configuration as the configuration with the highest performance, favoring fewer modalities in case of equality.

#### Segmentation uncertainty

The use of Monte Carlo dropout (MCDO) [30] allows the comparison of epistemic uncertainty between configurations, i.e., the uncertainty of the model, which can originate from its architecture and the representativeness of the training data set [31]. Since for each of the tested architectures our models only differ by the used modalities in training data, comparison of these uncertainties allows to assess how the presence or absence of particular input modalities influences the model’s certainty about its prediction.

The MCDO method includes using dropout during inference and producing multiple segmentation outputs for the same input. For each input, 30 samples were generated with a dropout rate of 0.3 for nnU-Net and 0.5 for SwinUNETR, as per recommendation from literature [30, 32]. For each voxel, the standard deviation over the predicted samples indicates a measure of uncertainty, from which uncertainty maps can be generated.

**Table 1:** Top performing configurations (according to mean Dice) per amount of input modalities

	Overall	ET	TC	WT
*nnU-Net*				
**1-input**	T1CE	T1CE	T1CE	FLAIR
**2-input**	T1CE-FLAIR	**T1CE-FLAIR**, T1-T1CE	T1CE-FLAIR	T1CE-FLAIR
**3-input**	**T1CE-FLAIR-T1**, T1CE-T2-FLAIR	T1CE-FLAIR-T1	**T1CE-FLAIR-T1**, T1CE-T2-FLAIR	**T1CE-T2-FLAIR**, T1CE-FLAIR-T1
*SwinUNETR*				
**1-input**	T1CE	T1CE	T1CE	FLAIR
**2-input**	**T1CE-FLAIR**, T1CE-T2	**T1CE-T2**, T1CE-FLAIR, T1-T1CE	**T1CE-FLAIR**, T1-T1CE, T1CE-T2	**T1CE-FLAIR**, T1-FLAIR, T2-FLAIR
**3-input**	**T1CE-T2-FLAIR**, T1CE-FLAIR-T1, T1-T1CE-T2	**T1CE-FLAIR-T1**, T1-T1CE-T2, T1CE-T2-FLAIR	**T1CE-T2-FLAIR**, T1CE-FLAIR-T1, T1-T1CE-T2	**T1CE-T2-FLAIR**, T1CE-FLAIR-T1, T1-T2-FLAIR

Multiple configurations are mentioned for statistically equal performances, with highest scoring mean denoted in bold

**Table 2:**
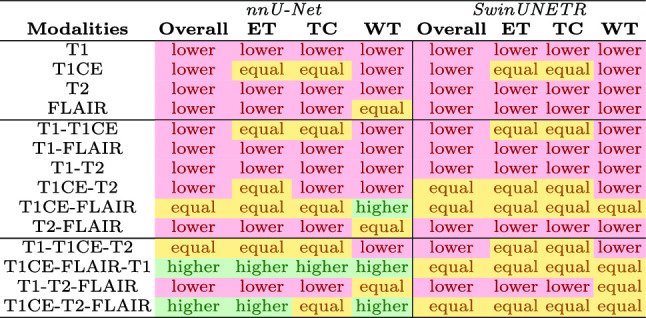
Segmentation accuracy performance in comparison with the full-input counterpart

**Table 3:** Minimal-input (MI) and optimal performance (OP) configurations (according to mean Dices)

	Overall	ET	TC	WT
*nnU-Net*				
**MI**	T1CE-FLAIR	T1CE	T1CE	FLAIR
**OP**	T1CE-FLAIR-T1, T1CE-T2-FLAIR	T1CE-FLAIR-T1, T1CE-T2-FLAIR	T1CE-FLAIR-T1	T1CE-FLAIR-T1, T1CE-T2-FLAIR
*SwinUNETR*				
**MI**	T1CE-FLAIR, T1CE-T2	T1CE	T1CE	T1CE-FLAIR
**OP**	T1CE-FLAIR, T1CE-T2	T1CE	T1CE	T1CE-FLAIR

Following [33], a subject-level uncertainty score for a model’s prediction was obtained by summing the standard deviations of all voxels, followed by division by tumor volume as to compensate for varying tumor sizes.

2 $$\begin{aligned} \textrm{Uncertainty} = \frac{\sum _{i}{\sqrt{\frac{1}{N}\sum _{n}(\hat{y}_{i,n} - {}\bar{\hat{y}_{i}})^2}}}{V}, \end{aligned}$$

where $\hat{y}_{i,n}$ is the prediction for voxel *i* in prediction sample *n*, $\bar{\hat{y}_{i}}$ is the mean prediction for that voxel, *N* is the total amount of samples and *V* is the tumor volume (i.e., the amount of voxels containing tumor).

Comparison of the configurations to the full-input configuration in terms of uncertainty then allows the determination of the minimal-input configuration.

#### Performance ranking

To simultaneously evaluate the different model configurations both in terms of segmentation accuracy and uncertainty, ranks for each model configuration were assigned per test subject. The higher the overall Dice score, the lower (i.e., the better) the ranking for segmentation accuracy. The lower the uncertainty score, the lower (i.e., the better) the ranking for uncertainty. Afterward, for each model, the ranks were averaged over all subjects.

#### Statistical analysis

To assess statistical significance, a Wilcoxon signed-ranks test with $\alpha =0.05$ was conducted when comparing models.

## Results

### Segmentation accuracy

Resulting Dice scores for the test set are summarized in Fig. 2. Top performing configurations per amount of input modalities, based on the mean Dice score, are summarized in Table 1. Multiple configurations are reported in case the differences were deemed statistically insignificant, of which the highest score is denoted in bold. All model configurations are compared to the full-input version in Table 2. Equal performance was reported when the comparison was found to be statistically insignificant. Significant differences in Dice scores were reported as either lower or higher accuracy. The minimal-input and optimal performance configurations are determined for each of the subregions and averaged over all subregions, and are reported in Table 3.

### Segmentation uncertainty

Resulting uncertainty scores for the test set are found in Fig. 2. Top performing configurations per amount of input modalities are summarized in Table 4. Only two cases were indicated statistically equal to the full-input model: T1CE-FLAIR-T1 and T1CE-T2-FLAIR for nnU-Net. In all other cases, uncertainty was found higher. Consequently, the minimal-input configurations according to mean uncertainty were found to be T1CE-FLAIR-T1 and T1CE-T2-FLAIR for nnU-Net and the full-input configuration for SwinUNETR.

**Table 4:** Top performing configurations according to mean uncertainty

	*nnU-Net*	*SwinUNETR*
**1-input**	**T1CE**, FLAIR, T2	**T1CE**, FLAIR
**2-input**	**T1CE-FLAIR**, T1CE-T2	**T1CE-FLAIR**, T1-T1CE
**3-input**	**T1CE-T2-FLAIR**, T1CE-FLAIR-T1	**T1CE-FLAIR-T1**, T1CE-T2-FLAIR

Multiple configurations are mentioned for statistically equal performances, with highest scoring mean denoted in bold

### Performance ranking

**Fig. 3:**
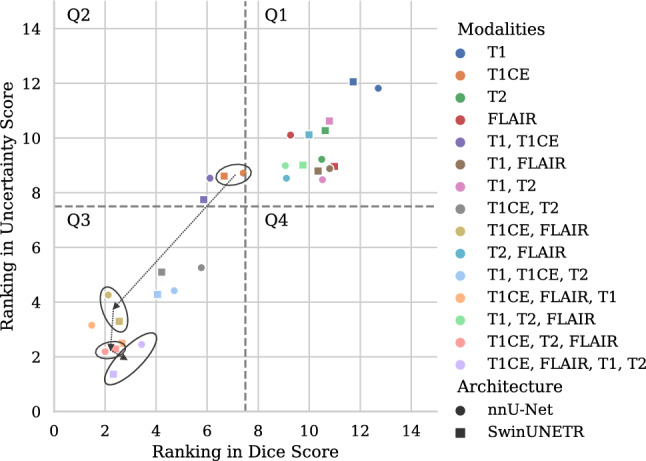
Performance ranking of models in terms of overall segmentation accuracy (Dice Score) and uncertainty (Uncertainty Score). For each group of models trained on the same amount of input modalities, the top performing model was selected as the model with the lowest Euclidean distance to the origin (in agreement between both architectures) and encircled. Arrows show the direction of an increasing number of modalities

Figure 3 summarizes the ranks in overall Dice score and uncertainties for the different model configurations. Lower ranks correspond to better scores; therefore, the closer a model is positioned to the origin of the graph, the higher its performance. We divide the graph in four quadrants (Q1–4), separating regions of high segmentation (Q2 and Q3) and high uncertainty performance (Q3 and Q4).

## Discussion

In this work, we evaluated 30 segmentation models trained with different input configurations and architectures in terms of segmentation accuracy and segmentation uncertainty and compare them to the full-input configuration.

The full-input models reached mean overall Dice scores of 0.79 for nnU-Net and 0.87 for SwinUNETR, which can be considered comparable with literature (0.85 for nnU-Net [27] and 0.89 for SwinUNETR [28]), taking into account that finetuning of our networks for optimal performance was beyond the focus of this work, and no ensembling from cross-validation, as conducted in [27, 28], was performed in order to reduce computational load. Comparison should also be made with caution since nnU-Net was trained on BraTS20 instead of BraTS21, and our models were trained on only a portion of the training set, while access to an additional validation set allowed the other works to fully utilize this set for training.

Considering the performance of models trained on reduced input, the following observations can be made (findings concern both architectures, unless explicitly stated otherwise). Segmentation accuracy results for the top performing single-input configurations (see Table 1) reveal the particular importance of T1CE and FLAIR imaging for automated GBM delineation. Table 2 shows that T1CE alone can suffice for the segmentation of ET and TC, yielding statistically equivalent results compared to the full-input version. Specifically for the nnU-Net architecture, the same holds for FLAIR-based segmentation of WT. Among two-input configurations, the T1CE-FLAIR emerges as a top performing configuration over all subregions and both architectures. This combination achieves equally high Dice scores for all regions compared to its full-input versions, and even higher scores for WT in the case of nnU-Net. Accordingly, the three-input configuration that exceeds in all cases consists of T1CE-FLAIR supplemented by T1. Compared to the full-input version, this configuration achieves higher scores for nnU-Net and equal scores for SwinUNETR over all regions.

From these results, we can conclude the configuration with the fewest amount of modalities that yields similar performance as the full-input configuration, which we refer to as the minimal-input configuration (see Table 3): T1CE alone is sufficient for the segmentation of ET and TC; FLAIR (nnU-Net) or T1CE-FLAIR (SwinUNETR) is sufficient for the segmentation of WT. T1CE-FLAIR is the minimal-input equivalent over all subregions. In other words, our results show that only T1CE and FLAIR are needed as input modalities to obtain Dice scores equal to utilizing the full input. When considering the optimal performance, we found for nnU-Net that not the full input, but a three-input configuration yields the highest Dice scores consistently over all regions, namely T1CE-FLAIR-T1. For SwinUNETR, said configuration shows equal performance to the full-input one. This indicates the possible confounding effect of redundant inputs mentioned in [18], for which nnU-Net appears to be more susceptible than SwinUNETR. We believe the multi-head attention mechanism of the latter allows for a stronger selection of relevant features in the early layers of the model compared to convolutions, making it more robust to input channel redundancy. However, this remains to be substantiated through designated experiments.

Epistemic uncertainty reflects the lack of knowledge of a model caused by limitations in its architecture and training data, making it suitable for evaluating the knowledge discrepancy of the models influenced by the presence or absence of particular input modalities. The preeminence of aforementioned configurations can also be discerned in such uncertainty-based analysis (see Table 4), where again T1CE and FLAIR; T1CE-FLAIR; and T1CE-FLAIR-T1 and T1CE-T2-FLAIR consistently come forth as optimal configurations for single-, two- and three-input categories, respectively. However, with the exception of T1CE-FLAIR-T1 and T1CE-FLAIR-T2 for nnU-Net, none of the cases demonstrate equal uncertainties to the full-input version.

Similar observations can be made through the analysis of the performance ranking (Fig. 3). Among the models in Q1, a common factor is the absence of T1CE, indicating the importance of this modality. T1CE, T1CE-FLAIR and T1CE-FLAIR-T2 show closest proximity to the origin for each of their input-number categories, signifying high performance. The addition of FLAIR to T1CE results in a substantial increase in performance rank. Further addition of T2 shows a more moderate improvement, which is nearly exclusively caused by movement in the uncertainty dimension, only showing insignificant change in Dice ranking. Lastly, the addition of T1 leads to minor improvement in the uncertainty dimension, while deteriorating the Dice ranking. Indeed, in Q3, the full-input models share their proximity to the origin with the T1CE-FLAIR, T1CE-FLAIR-T1 and T1CE-T2-FLAIR models, indicating their comparable performance. This implies that the addition of more modalities to T1CE-FLAIR ceases to increase the segmentation accuracy, while it causes a limited and increasingly smaller improvement in segmentation uncertainty.

Due to fundamental differences between the two architectures and their training, it is challenging to make a reliable comparison between both, in particular in terms of model uncertainty. This was however out of the scope of our study, as we primarily aimed to illustrate the generalizability of our findings over different model architectures. Indeed, overall similar trends are found both in Dice and uncertainty, most easily appreciable in Fig. 3, where corresponding data points of the different architectures are consistently found in close proximity to each other.

In essence, our findings suggest that the most essential information for this task is captured in T1CE and FLAIR. Incorporating additional modalities mainly reduces segmentation uncertainty. These findings align with our intuitive understanding, as the contrast-enhancing part is defined by contrast on T1CE and the hyper-intensity caused by edema is recognized on FLAIR. T1 and T1CE only differ by the addition of contrast in the latter. T2 and FLAIR share most of their characteristics, other than the signal suppression of cerebrospinal fluid in the latter. This may give rise to the presence of redundant information in the combination of these images, which does not contribute to or can even worsen segmentation performance. Moreover, these conclusions are in line with results from Bianconi et al. [34], which showed good segmentation results using only T1CE and FLAIR and no significant differences in performance when adding artificially synthesized T1 and T2 images, both for pre- and postoperative imaging. In addition, these results are consistent with those found using the modality importance (MI) metric defined in [14], which corresponds to clinicians’ prioritization of the modalities based on Shapley value. Considerably, higher MI values were reported for T1CE and FLAIR compared to T2 and T1, also corresponding to the importance accentuated by the saliency maps generated from their segmentation model. For nnU-Net, we found that a three-input configuration outperformed the full-input configuration, aligning with the findings in [18] that indeed the addition of nonessential modalities has the potential to worsen segmentation performance, although this does not extend to the other investigated architecture.

Several limitations to this study should be acknowledged. Firstly, only two state-of-the-art architectures were examined, representing convolution-based and transformer-based models. While we expect similar results, the generalization of these conclusions to other segmentation architectures remains to be confirmed. Due to the size and multi-institutional nature of the BraTS dataset, encompassing a vast range of imaging equipment, protocols and qualities, we anticipate a certain robustness of the networks to acquisition differences, but further validation on other datasets would be recommended. We acknowledge that the preprocessing of this dataset certainly has impact on the results, but left this unaltered because such standardization enables benchmarking of research and investigating the influence of specific preprocessing steps was beyond the scope of this study. We also note that our results might be influenced by the strong semantic correlation which exists between segmentation structures and the images they were annotated on, potentially increasing their importance for automated segmentation. As mentioned in [2], edema is segmented on T2 and afterward checked against FLAIR. Thereafter, the tumor core is segmented through the evaluation of T1CE hyper-intensities and T1 inhomogeneities. The enhancing tumor is then defined by thresholding the T1CE intensities within this core, while the necrotic core is located within this enhancing rim. This limitation is however inherent to manual segmentation labels and is challenging to overcome. Lastly, we note that while certain clinical indications may call for the acquisition of particular or more modalities, this work focused on the value of such images specifically in the context of automated GBM segmentation.

## Conclusion

In our study, we compared the performance of automated segmentation models, only differing by their amount and types of input modalities, and compared it to the performance of models trained on the full input (T1, T1CE, T2 and FLAIR) to gain insight on the importance of these modalities for the automated segmentation of GBM on MRI.

Our findings show that T1CE-based segmentation leads to equivalent accuracy for the delineation of ET and TC, compared to its full-input version. Adding FLAIR allows to achieve the same for WT and overall, suggesting the most essential information for automatic GBM segmentation is captured in T1CE and FLAIR, and T1CE-FLAIR-based models can be considered as a minimal-input alternative to the full-input configuration. The addition of modalities beyond this mainly lowers the segmentation uncertainty, but can also deteriorate accuracy. For nnU-Net, the segmentation accuracy was found to be significantly better for a three-input configuration (T1CE-FLAIR-T1) than when considering the full input.

## Footnotes

1 https://github.com/sdesutter/GBM-Segmentation-MRI.
